# A Review of Intraocular Pressure (IOP) and Axial Myopia

**DOI:** 10.1155/2022/5626479

**Published:** 2022-07-09

**Authors:** Dongyan Zhang, Liyin Wang, Le Jin, Yingying Wen, Xuhong Zhang, Liyue Zhang, Hong Zhu, Ziyu Wang, Xin Yu, Chen Xie, Jianping Tong, Ye Shen

**Affiliations:** Department of Ophthalmology, First Affiliated Hospital, College of Medicine, Zhejiang University, Hangzhou, Zhejiang 310003, China

## Abstract

The pathogenesis of myopia is driven by genetic and environmental risk factors. Accommodation not only alters the curvature and shape of the lens but also involves contraction of the ciliary and extraocular muscles, which influences intraocular pressure (IOP). Scleral matrix remodeling has been shown to contribute to the biomechanical susceptibility of the sclera to accommodation-induced IOP fluctuations, resulting in reduced scleral thickness, axial length (AL) elongation, and axial myopia. The rise in IOP can increase the burden of scleral stretching and cause axial lengthening. Although the accommodation and IOP hypotheses were proposed long ago, they have not been validated. This review provides a brief and updated overview on studies investigating the potential role of accommodation and IOP in myopia progression.

## 1. Introduction

Myopia, also known as “short-sightedness” or “near-sightedness,” is an increasingly widespread condition around the world, particularly in East Asia [1]. It has been predicted that by 2050, there will be approximately five billion people, equivalent to 49.8% of the world population, suffering from myopia [2]. Myopia is commonly defined as a spherical equivalent (SE) ≤ −0.5 dioptres (D) and develops mainly during childhood and early adulthood when excessive elongation of the eye causes images of distant objects to fall in front of the retina with the eye at rest, resulting in a blurred distance vision [3]. High myopia (SE ≤ −6.0 D) and pathologic myopia (pathological retinal changes secondary to high myopia) are more harmful than low-grade myopia, which may cause uncorrectable visual impairment or blindness, including sight-threatening diseases such as glaucoma, retinal detachment, and macular holes [4].

Refractive development has been shown to be affected by genetic factors and environmental risk factors [5]. Genetic linkage studies, exome sequencing, and whole-genome sequencing have identified some genetic factors associated with myopia [6, 7]. Environmental factors include educational attainment, near-work activities (reading, writing, watching TV, video, and other screens), sleep duration, and outdoor activities[1, 8]. Furthermore, gene-environment (*G* × *E*) interactions are also used to explain the problem of the contradiction between the limited genetic drift time and the rapid increasing incidence of myopia [3]. In the absence of the gene-environment interaction, there are independent effects between increased genetic risks of myopia and exposure to high-or low-risk environments. However, gene-environment interactions make a genetic variant strongly associated with certain environmental factors, that is, those with myopia susceptibility genes are more likely to develop myopia with the influence of certain environmental factors [3].

Several measures, including optical interventions, pharmacological interventions, and surgical treatment, have been established to correct blurred distance visions. Single-vision spectacles or contact lenses are the mainstays of managing myopia, which primarily correct the myopic refractive error. Refractive surgery is classified into keratorefractive procedures or intraocular procedures [9]. The former involves using an excimer or femtosecond laser to alter the curvature of the cornea, while the latter includes phakic intraocular lens (PIOL) implantation and cataract surgery to correct the refractive error [9]. However, most methods of correcting myopia do not address the underlying problem of elongation of the eye and do not suspend the pathological changes associated with high myopic progression. Wearing orthokeratology (OK) lenses have been shown to effectively slow down myopic progression, but they only work when worn overnight [10]. Additionally, a low dose of atropine, an effective nonspecific antimuscarinic antagonist, has been clinically used as a method for retarding myopia in myopic children and teenagers [11, 12]. It has been initially postulated that excessive ocular accommodation is the main cause of myopic progression, which is the premise upon which atropine functions [13]. Moreover, clinicians recommend that children should spend approximately two hours in an outdoor environment, which is necessary for retarding refractive changes and preventing myopic onset [14].

An abundance of theories has aimed at explaining the mechanism of the onset and progression of myopia. Animal models have shown that there probably is a growth-regulating cascade within the retina and the sclera, where multiple neural channels, neurochemicals, and their receptors have been implicated, such as the retinal ON-pathway and the regulation of dopamine [15–18]. It is also recognized that retinal visual growth signals are conducted by peripheral defocus and an accommodation lag, finally causing axial elongation [19, 20]. Based on these theories, multifocal soft contact lenses are specially designed to delay the progress of myopia [21]. The theory on the hypoxic microenvironment in the sclera was put forward recently since the investigators observed that the reductions in choroidal thickness and choroidal blood perfusion influenced by visual signals in myopes might cause scleral hypoxia related to the HIF-1*α* pathway [22]. It is universally acknowledged that extracellular matrix (ECM) remodeling in the sclera is a downstream event in myopia, resulting in a decline in scleral strength, scleral thinning, and axial elongation [23]. In spite of a large number of studies on myopia, the role of accommodation and intraocular pressure (IOP) in the potential biomechanical mechanisms of myopia has not been elucidated [1].

**Figure 1:**
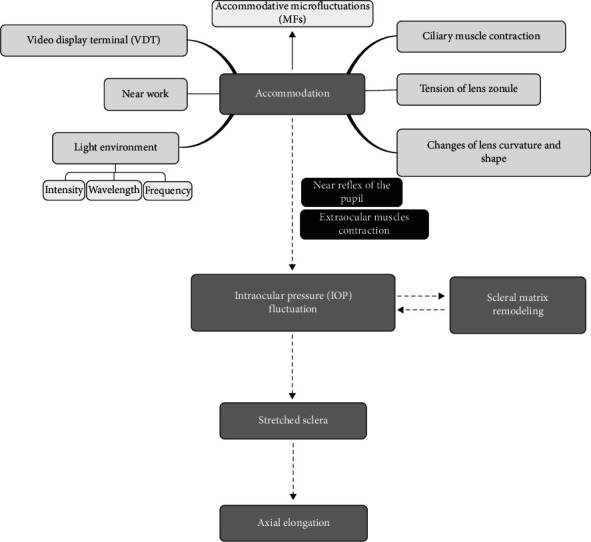
Overall framework of the hypothesis regarding accommodation-induced IOP fluctuations in myopia.

Traditionally, the persistent accommodation demands of greater near-work activity are probably associated with the underlying genetic susceptibility in myopia [24–27]. Anomalies of accommodative responses and reaction times during near work have been proposed as a causative factor in the development of myopia [28, 29]. During accommodation, the contraction and relaxation of the ciliary body cause changes in the shape of the lens, which affect the surface curvature and refractive power of the lens [30, 31]. During the high frequency of lens shape changes, some sustained pressure might be produced and conducted to the eyeball wall, which results in axial elongation. The near reflex of the pupil, consisting of accommodation, convergence, and miosis [32], should maintain a certain degree of coordination to ensure normal near-work activity. However, extra convergence in near work induces thickened extraocular muscles and elevated IOP, which is one of the common theories for the causes of myopia [33]. In addition, a recent cross-sectional observational study reported that intraocular pressure is positively correlated with high myopia [34]. Although it is not clear whether the change of IOP is the cause or result of myopia progression, IOP may be a cofactor, and it seems worthwhile to evaluate its possible role in axial myopia [35]. In this review, we focus on the role of IOP fluctuations and the potential biomechanical mechanisms of myopia (Figure 1); the myopia we mentioned mostly refers to axial myopia.

## 2. Accommodation in Myopia Development

As early as in the 20th century, the association between sustained near work requiring high levels of accommodation and the development of myopia has been well documented [36, 37]. Therefore, it was proposed that the increased accommodative effort required during near work might be a factor in myopic development [28]. Studies have shown that accommodative responses of myopic individuals may differ from those of their emmetropic counterparts [38]. Compared to the emmetropes or hyperopes, the accommodation lag at higher stimulus values was increased for the myopes [39]. Moreover, there was a strong correlation between refractive error and accommodative response gradient or tonic accommodation [39, 40]. Hyperopes had a higher response gradient than myopes [39]. Corrected hyperopes had a higher dioptric value of tonic accommodation and corrected late-onset myopes had a lower dioptric value [40]. The time taken to reach a stable tonic position of accommodation was also much slower for hyperopes than myopes [40]. Myopes whose eyes have a lower sensitivity to dioptric blur present less stable accommodation responses, and these inaccuracies of accommodation may cause long-term blurs on the retina, which is correlated with a failure of emmetropization [41]. Additionally, several dynamic accommodative response functions after near-work exhibited significant improvement subsequent to the accommodative training [42]. Therefore, it is conjectured that the accommodative facility might be a good predictor of future myopic progression.

Nearwork-induced transient myopia (NITM) has been proposed to describe the phenomenon of a temporary distant point getting closer and a transient myopic shift following a period of near work [26, 43]. The accommodation accuracy during near work is maintained by normal functioning of the ciliary body, lens zonules and crystalline lens, as well as complete autonomic nerve reflexes. Otherwise, the defocused retinal image will contribute to myopic development due to the accommodation lag [38]. Additionally, multiple clinical investigations have reported a temporary elongation in the ocular axis and changes in the ocular shape after accommodation (Table 1) [31, 44–47], which proved that abnormal accommodation in near work might be responsible for myopic progression [48, 49]. Prolonged accommodation caused axial elongation, while the eyeball shape was restored after rest. If the association between NITM and permanent myopia (PM) can be confirmed, the important role of accommodation in myopia can be further verified [50, 51]. Outdoor activities, which effectively reduce the demands of accommodation and relieve eye fatigue, are recommended by many ophthalmologists to alleviate myopia in children or young adults [52, 53]. It has been speculated that the mechanism by which outdoor activities delay myopic shift may be correlated with differences in luminous intensity and spectral composition between outdoor sunlight and indoor illumination environments, as well as outdoor relaxation of the ciliary muscle that ameliorates ciliary muscle spasms in excessive accommodation [54, 55].

Currently, the specific mechanism of excessive accommodation-induced transient myopia is yet to be determined. Read et al. [46] postulated that changes in the ocular structure such as scleral biomechanical properties in myopia might be attributed to the susceptibility of myopic eyes to accommodation-induced transient axial elongation observed in near work. Besides, the gaze angle was associated with short-term changes in axial length and ocular shape [44, 56]. Thus, Walker and Mutti [44] proposed that an expanded eye shape during accommodation might be caused by the transformation of eye position between primary gaze and peripheral gaze, resulting in increased tension of the extraocular muscles, oblique muscles in particular.

There also are voices questioning the role of accommodation as a major causative factor of myopia. McBrien et al. [57] found that the muscarinic antagonist atropine reduces experimental myopia and eye enlargement in chicks via a nonaccommodative mechanism. The most possible explanation is the difference of the anatomical structure and the mechanism of ocular accommodation between chicks and mammals [58, 59]. Myopia in chicks is caused by corneal curvature changes through the skeletal muscle [58]. While in mammals, the accommodation response is mainly controlled by the shape of the lens and the contraction and relaxation of the ciliary muscle (a smooth muscle). Therefore, the results in chicks may not be directly transferable to mammals. In conclusion, the role of accommodation in animals cannot be discounted. Accommodation induced by various methods may affect ocular shape and refraction through certain unknown pathways, which subsequently causes myopia. It is valuable and of great importance to establish reliable and intact methods of measuring ocular accommodation for future prediction of myopic children.

## 3. Intraocular Pressure Fluctuations and Accommodation

It has been proposed that intraocular pressure plays a crucial role as a mediator between accommodation and myopia. Young [60] implanted a sensor into the vitreous cavity of primate animals to directly measure the vitreous cavity pressure. When accommodation was induced in near work, the vitreous cavity pressure of primates increased [60]. For this reason, they postulated that an increase in fluid pressure enhanced irreversible elongation of the eyeball, following the changes repeated in the ciliary muscle during accommodation [60]. Jampel and Mindel [61] induced extraocular muscle contraction by stimulating the oculomotor center in monkeys, resulting in an increase in IOP. Therefore, they proposed that the elevated IOP was induced by accommodation through its related convergence in near reflex, that is, a compression pressure on the eyeball surface by extraocular muscle contraction [61].

Besides, recent studies have reported that accommodation can induce transient IOP elevation [62], simultaneously accompanied by declined anterior chamber depth, narrowed anterior chamber angle, and thickened lens thickness in progressing myopes and emmetropes [63]. However, there were no differences between baseline IOP and accommodation-induced IOP changes in myopes and emmetropes [62]. Ostrin and Glasser, and Abhiram and Glasser [64, 65] implanted an indwelling electrode in the Edinger-Westphal (E-W) nucleus of the mesencephalon in rhesus monkeys and induced accommodative responses by electrical stimulation of the E-W nucleus or pharmacological stimulation. They discovered that there were systematic linear correlations between dynamic accommodative refractive changes and biometric changes in anterior chamber depth (ACD), anterior segment length (ASL), and lens thickness (LT) in rhesus monkey eyes [65]. Moreover, the ciliary processes and the edge of the lens moved centripetally and linearly when accommodative refraction was changed by E-W nucleus stimulation [64]. That was to say, during accommodation, not only the ciliary muscle and the lens were changed, but also the biological length of the anterior chamber depth, the length of the anterior segment, and even the intraocular pressure might also be changed. Then, through pilocarpine stimulation, which was a nonselective cholinergic muscarinic agonist, Ostrin et al. [66] observed biological changes in decreased anterior chamber depth, increased lens thickness, and reduced pupil size coupled to myopic shift in the refractive error in a guinea pig.

A computer-simulated accommodation model supported the synergism of the ciliary body/zonule/anterior hyaloid complex, which promoted posterior lens surface changes during accommodation [67]. Coleman [68] presents a model which demonstrated the function of active vitreous support of the lens during accommodation, since the translational and irregular movement of the lens, that was, the front of the lens moved more than the posterior surface, was observed. Furthermore, fluid or hydraulic pressure gradients between the vitreous, lens, and anterior chamber were formed during accommodation [68]. Araki et al. [69] found using a fiber optic telescope that a contraction of the ciliary muscle does indeed produce “remarkable traction and advance” of the ora serrata. One study found that the mean amplitude of change in IOP over a 24-hour period was 3.12 ± 0.94 mm·Hg. When given an accommodative stimulus from 0 D to 6 D, the IOP significantly increased (1.02 ± 2.07 mm·Hg) in progressing myopes, but remained unchanged (mean change −0.76 ± 3.22 mm·Hg) in emmetropes [63]. Consequently, a series of changes during accommodation in the eye, including the changes of lens morphology, compression on the eyeball wall by contraction of the extraocular muscle and the ciliary muscle, and the formation of pressure gradients between the vitreous, lens, and anterior chamber, would lead to fluctuations of the IOP [60–63, 68, 69].

Therefore, accommodation leading to intraocular pressure variation might be one of the causative factors for axial lengthening resulting from the eyes focusing on a variable target, such as LED screens, for a long time in near work [70].

Nevertheless, intraocular pressure reduction was also observed to occur in accommodation [71–73], contrary to the abovementioned statement. A clinical study found that in patients with both myopia and emmetropia, IOP decreased significantly with increased accommodation [71, 73]. Moreover, IOP decreased the following alternating accommodation, but was not persistent in accommodation in healthy adult volunteers [74].

Contrasting findings from different studies imply the complexity of the relationship between ocular accommodation and intraocular pressure fluctuations, and probable reasons for the differences may be the asynchrony existing in pressure detection and accommodative stimulation. The concept of a pressure gradient between the vitreous and aqueous compartments of the eye can also explain the differential results of pressure measurements [68]. A reliable in-vitro experimental model in which the IOP can be rapidly manipulated, and can be simultaneously and continuously measured has been established to determine the physiological influence of intraocular pressure on the accommodative mechanism [75]. In summary, accommodation may contribute to pulsatile changes in IOP, and then stimulate longitudinal eye overgrowth in myopia [76]. The effects of ocular accommodation on IOP fluctuations are comprehensively influenced by multiple factors in biomechanics, and specific mechanisms should be further evaluated.

## 4. Intraocular Pressure and Myopia

It is widely believed that intraocular pressure, at a normal range of 10–21 mm·Hg, provides the growth signal and maintains eyeball integrity during eye development [77]. Elevated intraocular pressure is a major risk factor for glaucoma, which tends to occur in myopic patients [78, 79]. Studies have reported the risks associated with various glaucoma subtypes in all degrees of myopia, including low and high myopia [80], which provides the basis for evaluating the possible common mechanism controlling glaucoma and myopia.

**Figure 2:**
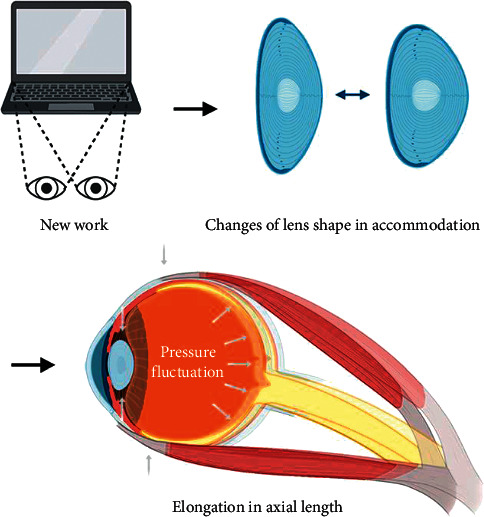
Axial elongation and IOP fluctuations induced by ocular accommodation and contraction of the intraocular and extraocular muscles in near work. Ocular accommodation in near work presents as changes in the lens shape as well as in the curvature and contraction of the ciliary muscles, accompanied by the contraction of the extraocular muscles in converge. Accommodation-induced IOP fluctuations are transferred from the liquid in the vitreous cavity to all directions, which possibly leads to eyeball deformation in the sagittal direction based on the susceptibility of scleral matrix remodeling in myopia.

It has been reported that there are correlations between changes in axial length and intraocular pressure fluctuations [81]. Both the axial length and IOP undergo significant variations over a 24-hour period, and they present a certain correlation [82]. Leydolt et al. [83] evaluated ocular biometric changes as a reaction to IOP changes in human eyes, and axial length elevation was observed when the IOP was increased by mechanical pressure on adult eyes. Moreover, the IOP level recovered and was accompanied by axial shortening after rest, thereby emphasizing the essentiality of measuring ocular rigidity in vivo in human eyes while detecting the IOP [83]. A study about the effects of high altitude on IOP and axial length found that a high altitude from Beijing to Lhasa led to a small but significant increase in IOP and axial length [84]. Exercise can also affect IOP and AL. After completing 10 minutes of moderate-intensity low-impact dynamic exercise, subjects experienced changes in ocular parameters, including a small but significant reduction in axial length, as well as decreases in IOP and ocular pulse amplitude [85]. These results suggested that IOP and axial length were jointly influenced by environmental factors, such as the aforementioned external pressure and exercise. In addition, one study included 397 eyes of 208 children, and the axial length data was plotted against the age. The LOWESS and quadratic fits were used to fit the nonlinear curve between the age and axial length with normal IOP (≤21 mm·Hg) and children with IOP >21 mm·Hg, respectively. The curve of IOP >21 mm·Hg is steeper than that of normal IOP at all the corresponding time points [86]. This means that with age, the axial length in children with high IOP rises more rapidly. Therefore, the axial length of healthy eyes increases with increases in IOP [83] and reduces with the reduction in IOP [85]. Pressure in the vitreous cavity which is transferred to all directions might lead to eyeball deformation in the sagittal direction (Figure 2).

In previous studies, the mechanism involved in myopia development was considered to be associated with scleral matrix remodeling [87], that is, the sclera becomes thinner in myopic patients [88, 89], especially in the posterior pole [90]; it is due to the degradation of the extracellular matrix (ECM) including collagen I, collagen III, collagen IV, and proteoglycan [91, 92]. This is often accompanied by an imbalance between matrix metalloproteinases (MMPs) [93–95] and tissue inhibitors of metalloproteinases (TIMPs) [96, 97], which cannot maintain normal extracellular matrix metabolism. In addition, thinning of the sclera and weakening of mechanical properties relate to the diameter of collagen fibrils, and studies have demonstrated that the collagen diameter in the outer layer of the posterior sclera was decreased in axial myopia [98].

A review of the relation between IOP, fundal stretching, and myopic pathology found some evidence of reduced collagen synthesis, altered collagen fibres, tissue loss, altered proteoglycans, increased matrix metalloproteinase activity, reduced scleral strength, and increased potential for creep (stretching or expansion) in response to the increase of IOP [99]. So the changes to the posterior fundus in myopia appear to be the consequence of mechanical tissue stretching and vascular changes which occur secondary to a process of fundal stretching due to axial elongation of the eyeball [99]. When IOP exerts a stretching tension on the outer scleral wall, eye elongation is more likely to occur in myopia due to scleral matrix remodeling, reduction in scleral rigidity, and decreased resistance to pressure [77, 100]. Therefore, a vicious circle forms [101], which might enhance susceptibility to IOP fluctuations induced by accommodation in the progression of axial myopia. Reducing exposure to the stress of elevated IOP appears to be a desirable form of intervention to control myopia, especially if myopic pathology can be reduced or prevented [99].

At the same time, various scholars have directly evaluated the relationship between intraocular pressure and myopia. Regarding whether the intraocular pressure hypothesis is relevant to the development of human myopia, the findings are contradictory (Table 2). Some studies reported that IOP is positively associated with high myopia [34, 102, 103], with IOP in the myopia group being higher than that before the onset [104]. In addition, a Japanese observational study reported that the prognostic factors for increased axial length include lower spherical equivalent, decreased choroidal thickness (CT), lack of the use of intraocular pressure-lowering medications, and other optic nerve and corneal factors [107]. Latanoprost, a prostaglandin analog that is effective in reducing IOP, was shown to significantly inhibit myopic progression in guinea pigs [100]. However, it has been reported that myopic progression over 2 years is inversely related to IOP [105], while other studies reported that there is no relationship between IOP and myopic progression as well as axial elongation [106]. So, there is no definitive conclusion on the relationship between intraocular pressure and myopia.

In fact, measurements of intraocular pressure are affected by various factors, including detection time, tonometer type, corneal thickness, corneal astigmatism, and other corneal biomechanical properties [108, 109]. Although Goldmann applanation tonometry (GAT) is the gold standard for IOP measurement [110], many other kinds of tonometers are still being used in various studies (Table 2), which limits data accuracy and comparisons of IOP levels among diverse studies to a certain extent. Measurements of tonometry also may be influenced by the axial length or a thinner and less rigid myopic posterior sclera [111]. When the scleral rigidity and resistance to IOP are reduced, the effect on the eyeball wall of a distending force of even low IOP is likely to be greater [112]. Furthermore, the IOP measured clinically is not the real IOP in daily life. Thereby, a tonometer for long-term detection of IOP to acquire reliable and periodic intraocular pressure levels is necessary for experimental and clinical work. Considering IOP measurements influenced by the possibility of biomechanical changes in both the posterior sclera and the cornea occurring with myopic progression, the comparison of results of longitudinal studies should be prudent.

In conclusion, we reviewed articles concerning the potential roles of intraocular pressure and accommodation in myopia progression. We acknowledged that accommodation and intraocular pressure fluctuations had a certain relationship through intraocular anatomy and biomechanical effects, and intraocular pressure fluctuations have played an important role in the development of myopia through scleral matrix remodeling.

## 5. Recommendations

Which comes first? IOP or myopia? although there is no reliable conclusion on this issue, these projects would have the following priorities to consider for investigators: (1) Develop a novel and noninvasive tonometer to monitor real-time intraocular pressure in the ophthalmologic field. While stimulating accommodation, real-time intraocular pressure monitoring will help us to perform curve fitting on accommodation and IOP and further understand the relationship between accommodation and IOP. (2) Apply the latest and developing techniques to establish an experimental model of accommodation and IOP manipulation. The current model is limited to the *in vitro* eye; if the model construction of the *in vivo* eye can be carried out, it will promote the explanation of this problem. (3) Initiate a multicenter, double-blind, prospective clinical trial of treatment with drugs to lower IOP and explore the regulation of drugs on progressive myopia in adolescent subjects. Directly exploring the effect of lowering intraocular pressure on the prevention and control of myopia can better explain the role of intraocular pressure in the development of myopia. The premise of this study is to control the adverse effects of IOP-lowering drugs on appropriate adolescent population with myopia.

## 6. Conclusion

The eyeball is a complex, rigid, and near-ellipsoidal liquid system. Ocular accommodation might induce intraocular pressure fluctuations through changes of the ciliary muscle and extraocular muscles in near work. Scleral matrix remodeling enhances susceptibility to intraocular pressure fluctuations in myopes, resulting in elongation of axial length in the sagittal direction. These biomechanical factors would promote myopia progression.

## Figures and Tables

**Table 1:** Accommodation-induced elongation of axial length in different refractive status.

Ref.	Year	Accommodation demands	Subjects	Refractive status	Ocular shape changes	Difference between myopia and emmetropia (increased axial length)
[31]	1998	2–20 D	23 young adults	Myopia and emmetropia	Axial elongation	More elongation in emmetropes than in myopes (5.2^*∗*^ vs. 12.7^*∗*^ *μ*m)
[44]	2002	0 D and 3 D	41 young adults	Relative peripheral refractive error (RPRE)	More prolate ocular shape	—
[45]	2006	0 D, 2 D, 4 D, and 6 D	60 young adults	Myopia and emmetropia	Axial elongation	A significantly greater transient increase in axial length in myopic subjects (58^*∗*^ vs. 37^*∗*^ *μ*m for 6 D)
[46]	2010	0 D, 3 D, and 6 D	40 young adults	Myopia and emmetropia	Axial elongation	No significant difference (11.2 ± 12.2 vs. 12.6 ± 12.8 *μ*m for 3 D; 23.1 ± 22.7 vs. 25.2 ± 15.0 *μ*m for 6 D)
[47]	2017	0 D, 3 D, and 4.50 D	72 subjects, aged 18–60 years	Myopia and emmetropia	Axial elongation	No significant difference (2 ± 18 *µ*m for 3 D; 8 ± 16 *µ*m for 4.50 D)

Data are presented as mean ± SD. ^*∗*^Mean.

**Table 2:** Relationships between IOP levels and refractive errors in cross-sectional and longitudinal studies.

Ref.	Year	Type of study	Subjects	IOP measuring instruments	Groups	Comparison	*p* value
[34]	2019	Cross-sectional study	6101 participants, aged ≥40 years	An auto refractometer (Tonoref II, Nidek, Gamagori, Japan)	Non-high myopia and high myopia	IOP (13.3^#^ vs. 14.3^#^ mm·Hg)	<0.001
[102]	1995	Cross-sectional study	321 children	A pneumatonometer (Digilab model 30R, Cambridge, MA)	Nonmyopia and myopia	IOP (17.4 ± 4.1 vs. 17.8 ± 3.5 mm·Hg, OD, 17.0 ± 3.7 vs. 17.9 ± 4.3 mm·Hg, OS)	<0.1 (OD) <0.05 (OS)
[103]	1992	Longitudinal study over 2 years	49 children, aged 9–12 years	Goldmann applanation tonometer	IOP ≤16 mm·Hg and IOP >16 mm·Hg	Refraction progression (0.86 ± 0.55 vs. 1.32 ± 0.7 D)	<0.05
[104]	1996	Cross-sectional and longitudinal study	106 children, aged 7–9 years	A pulsair noncontact tonometer	Pre-existing myopes, incident myopes, and control	IOP (15.17 ± 3.54 vs. 13.88 ± 2.85 vs. 13.43 ± 1.88 mm·Hg)	>0.05
					Incident myopes and before	IOP (mean difference 1.19^*∗*^ mm·Hg)	<0.05
[105]	2019	2-year longitudinal study	1558 grade 7 students, aged 12 years	A noncontact tonometer (HNT-7000, Huvitz)	Refraction progression ≥1 D and <1 D	IOP (15.69^*∗*^ vs. 16.09^*∗*^ mm·Hg)	<0.05
[106]	2008	5-year longitudinal study	104 children, aged 6–11 years	Tono-Pen XL (mentor ophthalmic)	High IOP (≥15 mm·Hg) and low IOP (≤14 mm·Hg)	Fast myopic progression (≥−1.75 D) and slow myopic progression (≤−1.625 D)	>0.05

Data are presented as mean ± SD. ^#^Median; ^*∗*^mean.
